# *It would be really great if I had greater confidence and ability there:* Understanding the primary care physician experience with nutrition in training, clinical practice, and interprofessional collaboration

**DOI:** 10.1371/journal.pone.0354813

**Published:** 2026-07-31

**Authors:** Caitlin A. Hildebrand, Seema Agrawal, Jaclyn Albin, Gary L. Beck Dallaghan, Elizabeth Chen, David Gaviria, Kurt O. Gilliland, Alison Hilton, Thomas C. Keyserling, Elizabeth Mayer-Davis, Alice S. Ammerman

**Affiliations:** 1 University of North Carolina at Chapel Hill, Nutrition Department, Chapel Hill, North Carolina, United States of America; 2 University of North Carolina Center for Health Promotion and Disease Prevention, Chapel Hill, North Carolina, United States of America; 3 University of North Carolina Cecil G. Sheps Center for Health Services Research, Chapel Hill, North Carolina, United States of America; 4 Departments of Pediatrics and Internal Medicine, University of Texas Southwestern Medical Center and O’Donnell School of Public Health, Dallas, Texas, United States of America; 5 Carle Illinois College of Medicine, University of Illinois Urbana-Champaign, Urbana, Illinois, United States of America; 6 Department of Health Behavior, Gillings School of Global Public Health, The University of North Carolina, Chapel Hill, North Carolina, United States of America; 7 Department of Cell Biology and Physiology, School of Medicine, University of North Carolina at Chapel Hill, Chapel Hill, North Carolina, United States of America; 8 Connected Health Applications & Interventions (CHAI) Core, Lineberger Comprehensive Cancer Center and Nutrition Obesity Research Center, University of North Carolina at Chapel Hill, Chapel Hill, North Carolina, United States of America; 9 Department of Medicine, School of Medicine, University of North Carolina, Chapel Hill, North Carolina, United States of America; Nathan S Kline Institute, UNITED STATES OF AMERICA

## Abstract

**Background:**

Primary care physicians (PCPs) are on the front lines of health promotion and disease prevention, yet evidence supports a longstanding gap in physicians’ capacity to counsel about diet, a key factor in patient health and chronic disease risk. Momentum to address the nutrition training needs of physicians is growing; however, challenges PCPs experience due to inadequate training are further complicated by clinical practice barriers. This study seeks to better understand the complex problem PCPs face addressing nutrition with patients to inform potential solutions.

**Methods and findings:**

This qualitative study takes a design thinking approach to understanding the problem by learning from PCPs about their experience, challenges, and priorities addressing nutrition with patients. Findings from semi-structured interviews with 32 PCPs revealed that while they value nutrition and believe they have an important role in addressing diet with patients, their role as a generalist in addressing nutrition lacks clarity. PCPs describe learning their current approach to nutrition primarily outside of formal medical training, resulting in variability and inconsistency in approaches and recommendations and general frustrations about their ability to give actionable guidance. While registered dietitians (RDs) are often viewed as valuable support for patients, PCPs describe multiple challenges to RD utilization and subsequently at times may be the only ones with the opportunity to address diet with patients. Additional system- and societal-level barriers, such as time limitations and the food environment, further complicate PCPs’ experiences advising patients about nutrition. Main limitations of this study are limited generalizability from recruiting graduates of a single medical school and PCPs with greater interest in nutrition or more frustration with their current approach to nutrition may have been more likely to participate.

**Conclusions:**

Our results support the need to clearly define a realistic role for PCPs as generalists addressing nutrition. Clarified expectations in turn can inform nutrition training of physicians and guiding frameworks and strategies for giving practical, evidence-based nutrition guidance. PCPs also need increased support through access to resources, strategies to improve RD collaboration, and attention to system- and societal-level challenges influencing dietary interventions in primary care.

## Introduction

Dietary behaviors are an important risk factor for multiple common chronic diseases regularly managed by primary care physicians (PCPs) [1,2]. Guidelines from the US Preventive Services Task Force and professional societies emphasize the importance of physicians offering or referring patients for dietary counseling to address chronic disease risk [3–6]. PCPs specifically are well-situated to address diet to promote health and reduce chronic disease risk, and studies suggest PCPs can motivate dietary change, even through a brief intervention or endorsement [7,8].

Despite their influential role, physicians historically have not been adequately trained in dietary counseling, and calls to reform nutrition training in medicine have gained momentum [9–14]. Beyond training deficiencies, PCPs report barriers to addressing diet in clinical practice, such as time constraints, inadequate reimbursement, and lack of resources, with little evidence of improvement over more than two decades [15–18]. Additionally, studies document an incongruence between physicians valuing nutrition yet reporting a deficiency in implementation of dietary counseling in practice [16,19,20].

As end users reflective of the spectrum of medical training and clinical practice experience, PCPs contribute an important perspective on nutrition training and patient care needs informed by real-world practice. However, large surveys assessing PCPs’ perceptions about nutrition counseling were conducted over two decades ago and are limited in depth of understanding [15,19,21]. More recent studies exploring this phenomenon have been limited by practice settings, medical conditions, or patient population [17,22–29]. Thus, an updated and comprehensive understanding of the general needs of PCPs to more effectively address nutrition is needed to identify strategies to support them in practice.

Wicked problems such as this require innovative methods to produce effective solutions. Widely used in the private sector, design thinking draws on the perspectives of the target population, or end users, to design solutions to dynamic, multifaceted problems. Design thinking promotes first understanding a challenge from an end user’s perspective, then leveraging a set of methods to ultimately create specific end user-informed solutions. Studies indicate design thinking use in health care interventions may result in more usable and effective interventions [30,31]. More specifically, exploring the end-user perspective of PCPs on addressing nutrition could lead to solutions clinicians will find helpful and regularly use, potentially increasing uptake in practice.

To address this complex gap in the literature and begin the journey toward solution-creation, this qualitative study uses a design-thinking lens to comprehensively explore PCPs’ experiences and challenges addressing nutrition in their current practice. This update to outdated literature and comprehensive exploration of real-world practice needs also add a valuable perspective on how to train physicians, especially given newly introduced accreditation standards for nutrition training in medical education, variability in nutrition curricula integration in medical schools, and gaps in understanding of strategies for vertical integration of nutrition in medical education [32,33]. Additionally, while medical trainees offer a valuable viewpoint on training needs [34–36], the PCP lens reflects how these gaps intersect with the realities of clinical practice demands and the available support through resources and referrals. Thus, the aim of this study is to initiate the design thinking process through an in-depth exploration of the experiences and challenges PCPs face addressing nutrition.

## Methods

### Study design

This study incorporated initial steps of a design thinking framework [37] to better understand the needs of PCPs in addressing nutrition through semi-structured interviews. The first phase of the design thinking process is centered around the problem space and understanding the lived experiences of end users. Interviews were conducted to gain in-depth knowledge of PCPs’ experiences addressing nutrition in their current practice and their experience training in nutrition. Given overlapping needs of PCPs caring for adults, pediatrics, or both patient populations and that the foundational Step 1 US Medical Licensing Examination (USMLE) includes knowledge of nutrition across the life course [38], this study includes perspectives from physicians in pediatric, adult, and full spectrum primary care practice. After data were collected, they were analyzed during the second step of the design thinking process to articulate an updated challenge.

### Design thinking approach

**Fig 1:**
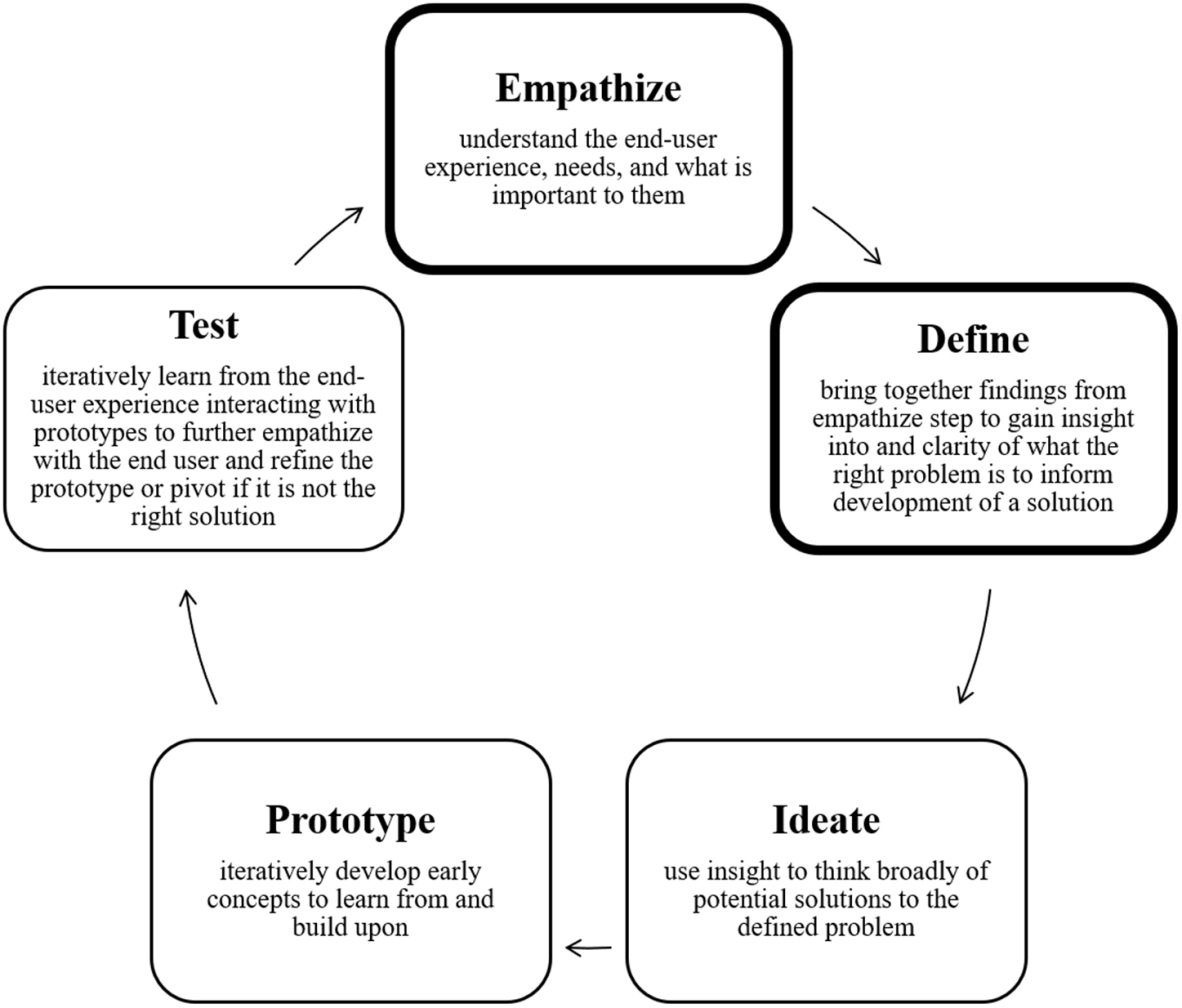
Steps of the design thinking process. The iterative steps of the design thinking process developed by the Hasso Plattner Institute of Design at Stanford [37] are represented and summarized. The first two steps of empathizing and defining the problem are in bold to highlight the steps undertaken in this study.

Design thinking, a form of human-centered design, is a useful strategy promoting innovation to solve complex challenges [30,39]. Aligning with a pragmatic epistemological approach, this qualitative study focused on the problem definition portion of the design thinking process by completing the first two steps: (1) empathize with the end user to understand their experience, challenges, and frustrations and (2) define the problem PCPs face addressing nutrition in their current practice [31,37,40]. Fig 1 gives an overview of the five steps and highlights in bold those undertaken in this study.

### Recruitment and participant characteristics survey

Our aim was to recruit currently practicing PCPs trained in Family Medicine, Internal Medicine, Pediatrics, or combined Internal Medicine/Pediatrics. Beginning with a list of University of North Carolina (UNC) School of Medicine (SOM) graduates (classes of 2012–2020), the principal investigator (CH) reviewed available information from residency match lists and publicly available information regarding medical specialty/field and reduced the list to decrease emails sent to those specializing in ineligible fields. An email with a request to participate and a link to complete a brief survey was sent to 412 email addresses. The recruitment email conveyed the desire to learn about their experience with nutrition and their patients to inform practical strategies for busy clinicians and to improve nutrition training of physicians at UNC and other programs.

The survey, administered using REDCap, began with an eligibility screening question followed by the consent form. Eligible participants who consented to participate were directed to a 12-item participant characteristics survey. To provide a context of participants’ backgrounds and clinical practice and to aid with purposive sampling if needed, the 12 items asked questions regarding demographics, clinical practice characteristics, adequacy of nutrition training, and confidence addressing nutrition. Recipients were invited to share the recruitment email with colleagues. We chose to recruit a sample closer to completion of medical training for increased recollection of their formal nutrition training and to reflect more recent trends in medical education. Initial emails were sent August 23, 2023, and up to five reminder emails were sent, the final sent February 9, 2024. Interviewees were offered a $30 gift card.

We initially aimed to complete up to 25 interviews or until thematic sufficiency, determined by the interviewer’s judgment on whether interviews continued to generate new experiences and insights. Due to the breadth of experiences, variety of patient populations and practice settings, and the duration of interviews, we interviewed as many PCPs as possible and conducted more interviews than originally intended. All participants who completed the scheduling process were interviewed to increase the variety of perspectives. The UNC Institutional Review Board granted this study exempt status (#22–1304).

### Interviews

The principal investigator (CH) conducted semi-structured interviews with 32 PCPs (Family Medicine, Internal Medicine, Pediatrics, or combined Internal Medicine/Pediatrics) from September 2023 to March 2024. Modeled after design thinking problem interviews, the interviews explored three broad questions regarding PCPs’ experience addressing nutrition in their clinical practice: current situation (e.g., experience with assessment, counseling, referrals), pain points (e.g., challenges, frustrations), and ideal situation (e.g., what they would wish for if they had a “magic wand”). The interview guide was organized by these categories with related prompts, including a prompt asking participants to share their experience learning nutrition. To reduce participant burden and to accommodate busy schedules of clinicians, interviews were designed to be brief, around 20 minutes; however, participants were given the option to extend the interview. Interview durations ranged from 18 to 53 minutes, and the average duration was 28 minutes. Interviews were conducted using a secure, HIPAA-compliant Zoom platform. Participants had the option to join by video or audio depending on preference. The interviewer conducted interviews in a private space, and participants elected their location when joining the interviews.

### Analysis

**Fig 2:**
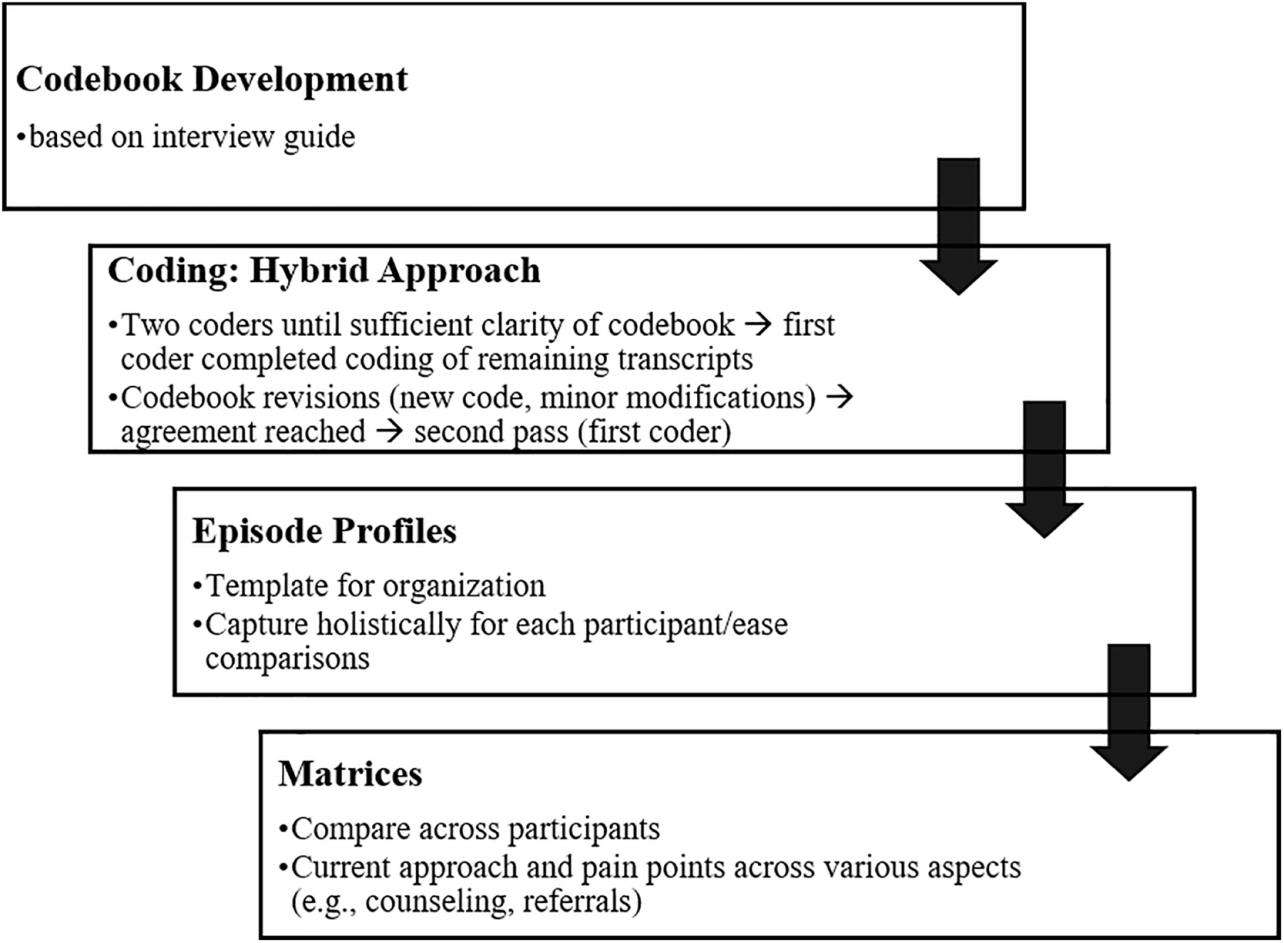
Overview of qualitative analysis steps. This figure maps the steps taken to code and organize data from interview transcripts.

Interviews were transcribed using Zoom transcripts or a transcription service. Transcripts were compared to audio recordings and corrected by the principal investigator (CH). An overview of steps in the analysis of qualitative data is outlined in Fig 2. The codebook used structural codes based on the interview guide as well as codes that emerged from the interviews. Using Dedoose software [41], two coders (CH and AH) independently coded transcripts until the two coders agreed the codebook had sufficient clarity in the code definitions and decision rules. The first coder (CH) completed coding of the remaining transcripts, consulting the second coder (AH) when needed. A new code was added based on a generated theme, and minor codebook modifications were made for greater clarity and uniformity. The two coders met to review and discuss codebook revisions, and, after agreement was reached, the first coder completed a second review of all transcripts. To organize coded data to reflect current situation, pain points, and ideal situation for each participant, episode profiles of participants were used. To create individual participant episode profiles, a summary template was developed, using the codebook as a guide. Episode profiles allowed for capturing individual participant experiences holistically while also allowing for ease of comparison between participants [42]. For comparisons between participants, matrices were created based on current approach to addressing nutrition and pain points for various aspects of their experience (e.g., assessment, counseling, referrals). Data from matrices were summarized and thematically analyzed through content analysis, creating outlines of data from each matrix and generating themes in the process. Illustrative quotes were selected. Background survey data were quantitatively analyzed using descriptive statistics. Mean confidence and standard deviation were calculated using SAS 9.4 software. Given the breadth of the data, this manuscript focuses on results and analysis for the first two steps of the design thinking process (Fig 1). After analysis of individual components of PCP experiences addressing diet (e.g., assessing diet, counseling, resources/referrals), and associated challenges, data was analyzed to identify key problems. Due to overlap between various challenges identified, a conceptual model of the identified problems was generated to understand and present the problems and how they relate.

### Positionality and reflexivity

The principal investigator (CH) led the inception, implementation, and analysis of this study while completing doctoral training in nutrition and brings personal and professional experience in medical training. Team members engaged in the development and refinement of the interview guide (CH, EC, AA) bring lived experience of medical training, patient care, interprofessional collaboration, patient barriers, and/or human-centered design. The principal investigator (CH), who conducted the interviews, has engaged in nutrition curriculum development and implementation at UNC SOM but this work did not overlap with the participants’ training. The combination of the principal investigator’s personal experience training in medicine and nutrition while not having lived experience as an independently practicing primary care physician brought empathy and curiosity to the interviews. The two coders (CH, AH) offered varying perspectives, one (CH) with expertise and lived experience relevant to the study and the second (AH) with expertise in qualitative methods applied in a variety of public health capacities, to discussions about the codebook and coding of transcripts. Multiple team members have expertise in applied nutrition research in clinical settings and some team members have experience implementing nutrition training in medical education.

## Results

### Participants

Of 59 initiated recruitment surveys, 52 were completed. Of those, 47 were eligible and consented to participate. Thirty-two PCPs completed the scheduling process and interview. Table 1 outlines participant characteristics for the final sample of 32. Fifty percent of participants were family physicians, and nearly half reported seeing adult and pediatric patients. Half of the sample practiced in North Carolina, and participants reported a variety of practice settings. Residency graduation years ranged from 2015 to 2023. All participants were UNC SOM graduates.

**Table 1:** Participant characteristics. Table 1 lists results from the survey completed by interview participants and includes demographics, training, practice characteristics, and confidence addressing nutrition. Abbreviations: Med-Peds (combined Internal Medicine/Pediatrics).

Participant Characteristics (n = 32)^a^	
**Demographics**	
**Gender** n (%)	**Female:** 17 (53%)
	**Male:** 15 (47%)
**Race** (all that apply) n	**Asian:** 1
	**Black or African American:** 1
	**White:** 29
	**Unknown:** 1
**Ethnicity** n (%)	**Hispanic or Latino/a:** 2 (6%)
	**Not Hispanic or Latino/a:** 30 (94%)
**Training Background**	
**Residency training**	**n (%)**
Med-Peds	4 (13%); (Adults only: 1; Adults and Pediatrics: 3)
Family Medicine	16 (50%); (Adults only: 5; Adults and Pediatrics: 11)
Internal Medicine	6 (19%)
Pediatrics	6 (19%) (Pediatrics only: 5; Adults and Pediatrics: 1)
**Year graduated residency**	**Range:** 2015–2023
	**Frequency (n):** 2015 (n = 6); 2016 (n = 3); 2017 (n = 4); 2018 (n = 3); 2019 (n = 4); 2020 (n = 1); 2021 (n = 9); 2022 (n = 1); 2023 (n = 1)
**Primary Care Practice Characteristics**	
**Patient population** n (%)	**Adults only:** 12 (38%)
	**Pediatrics only:** 5 (16%)
	**Adults and Pediatrics:** 15 (47%)
**Where currently practice** n (%)	**NC:** 15 (47%)
	**Outside NC:** 15 (47%)
	**Unknown:** 2 (6%)
**Location** n (%)	**Urban/large city:** 11 (34%)
	**Suburban:** 10 (31%)
	**Town/small city:** 9 (28%)
	**Rural:** 2 (6%)
**Practice type** n (%)	**Private practice:** 11 (34%)
	**Academic/university-affiliated:** 7 (22%)
	**Community hospital-affiliated:** 6 (19%)
	**Federally qualified health center (FQHC):** 5 (16%)
	**Veterans Affairs clinic:** 1 (3%)
	**Public health department:** 0 (0%)
	**Other:** 2 (6%)
**Approximate percentage of patients that are uninsured or receive Medicaid** n (%)	**No patients:** 3 (9%)
	**Between 0–25% of patients:** 17 (53%)
	**25-50% of patients:** 6 (19%)
	**50-75% of patients:** 2 (6%)
	**>75% of patients:** 4 (13%)
**Nutrition Training**	
**Adequacy of training received** n (%)	**Inadequate:** 20 (63%)
	**Adequate:** 11 (34%)
	**More than necessary:** 0 (0%)
	**None:** 1 (3%)
**Confidence Addressing Nutrition with Patients**	
**Confidence level** n (%)	**5, Completely confident:** 1 (3%)
	**4, Fairly confident:** 11 (34%)
	**3, Somewhat confident:** 13 (41%)
	**2, Slightly confident:** 5 (16%)
	**1, Not confident:** 1 (3%)
	**Unknown:** 1 (3%)
**Mean Confidence (**n = 31)	3.19 (SD = 0.87)

^a^For items with missing data, sample sizes are provided with the item.

### Step one: Empathizing with PCPs

Results from the empathize step (Fig 1) are organized below into three categories: participants’ experiences learning nutrition, their current approach to nutrition, and perceptions about nutrition in primary care.

### Experiences learning nutrition

In general, PCPs described deficiencies in formal nutrition training during medical school and residency. Nutrition was often characterized as “not a focus,” such as a brief mention of a recommended diet at the end of a lecture. Some had difficulty remembering nutrition training.

*“I can’t remember it in med school…it’s not really coming back to me as like a specific block or something. I feel like probably it was kind of sprinkled in throughout the different curriculum of like cardiology, endocrine…”*

PCPs described inadequacies in their nutrition training not only in the amount but also in the content covered. The nutrition they learned and were tested on was not necessarily what they need on a daily basis in clinical practice. While medical school nutrition content was often described as unmemorable, what was remembered was learning micronutrients, particularly vitamins, and information not useful to their practice.

*“…they did a really nice job of explaining what vitamin C does and what magnesium does…there wasn’t good translation to tell the patient what to do…I felt really, really well-equipped to deal with I’m gonna say sick people. But…talking to patients about how to make healthy choices has been much more difficult.”*

Multiple PCPs described receiving minimal to no formal or standardized nutrition training during medical school and residency. Many described subsequently learning nutrition on their own, mostly through self-directed or independent learning, particularly in response to “on the job” questions or knowledge gaps. A common gap described was lack of training in specifics of what to tell the patient or lacking in actionable guidance. It was uncommon for a PCP to describe feeling satisfied with their formal nutrition training during medical training. Interactions with registered dietitians (RDs), infrequently mentioned as an educational resource during training, were mostly informal or elective experiences. Participation in continuing medical education nutrition trainings was uncommon, and some participants described them as failing to meet their needs. A limited number of participants completed nutrition-relevant certifications (e.g., Lifestyle Medicine, Obesity Medicine), with one commenting that prior to this training they only felt equipped to tell patients to “eat less and exercise more.” Given how often nutrition comes up, participants described a mismatch in what patients expect from them and their training.

*“…one of the reasons that I…[did] this meeting with you…was just really thinking about how much this counseling comes up on a day to day…basis for me. And again, feeling like, wow, there’s just, there’s so much that it feels like patients see us as authorities to talk through. But yet it’s not necessarily what we’ve been trained to do…”*

Most participants reported being somewhat (41%) or fairly (34%) confident addressing nutrition with patients (Table 1). Some areas PCPs described lacking comfort in addressing nutrition included the following: counseling on specific diet plans, giving concrete/practical dietary guidance, identifying problem areas, disordered eating counseling, and weight counseling/fear of causing harm through weight discussion.

### Experiences addressing nutrition in current practice

**Fig 3:**
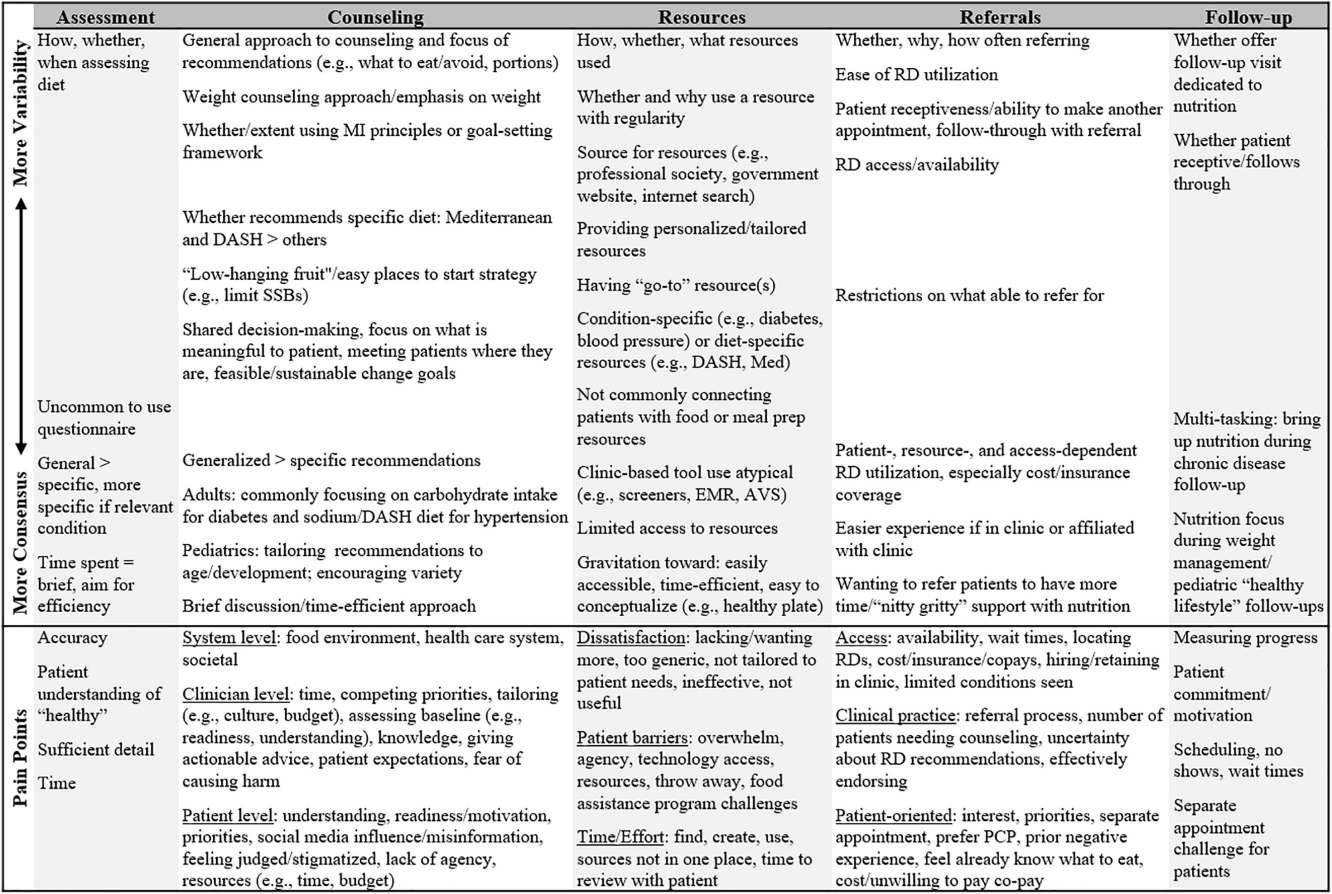
Overview of PCP participants’ current approaches to nutrition and pain points. This figure maps five elements of addressing nutrition in primary care, beginning with dietary assessment and followed by counseling, resource use, referral-making, and nutrition-relevant follow-up care. The top section is relatively ordered by variability versus consensus, with more typical practice toward the bottom and greater variability in practice toward the top. Pain points (e.g., challenges, frustrations) across each of the categories are outlined in the bottom portion of the figure. Abbreviations: AVS (after visit summary), DASH (Dietary Approaches to Stop Hypertension), EMR (electronic medical record), Med (Mediterranean), MI (motivational interviewing), PCP (primary care physician), RD (registered dietitian), SSB (sugar-sweetened beverage).

Capturing multiple aspects of PCPs’ experiences addressing nutrition in their practice, the following results report on prioritization of nutrition in patient encounters, reasons nutrition is discussed in primary care, PCP approaches to addressing nutrition during patient encounters, challenges and frustrations PCPs experience addressing nutrition, and their experience with nutrition support and services. To highlight PCP experiences within and across patient encounters addressing nutrition personally and collaboratively, Fig 3 provides an overview of participants’ experiences across five elements of addressing nutrition with patients: dietary assessment, counseling, resources, referrals, and follow-up visits. Fig 3 also presents the multiple participant approaches and experiences through relatively ordering by variability among participants, with greater variability at the top and more consistency at the bottom. While challenges and frustrations are presented throughout the following results, Fig 3 provides a summary of pain points (e.g., challenges, frustrations) for each corresponding category.

#### Prioritization of nutrition.

Prioritizing nutrition in practice was mixed, with some describing addressing nutrition with regularity, often dependent on the visit type or patient history. Even if addressing nutrition regularly, many described a limited amount of time spent (Fig 3). Competing priorities and health system constraints were predominant challenges that influence whether nutrition is addressed and the amount of time spent, with PCPs often describing having to fit in multiple tasks in a short visit (Fig 3).

*“The number one issue is, like I’ve mentioned several times, is time…most of my visits are about 15 minutes. That also includes time with the patient, plus time that you’re supposed to write the note, put in orders, send medications, and it’s laughably impossible to do all of that. But that’s the way the system is currently.”*

Uncommonly PCPs described intentionally starting with nutrition to emphasize importance. More often PCPs commented they try to address nutrition, but it frequently gets “glossed over” or “pushed down the priority ladder” due to time constraints and competing priorities. Some PCPs’ decision to address nutrition was based on patient body mass index (BMI).

*“…if I was doing the best medicine I could…I’d be talking about nutrition…with pretty much all my patients, but I find myself doing it in a very reactionary type manner. So, if I notice someone’s overweight or underweight or obese…that’s mostly the times I find myself talking about it.”*

Perceptions about brief dietary discussions varied. Some felt time constraints limited their ability to help patients make meaningful changes. On occasion, PCPs expressed concern that brief discussions were “not doing the patient any favors” or could unintentionally contribute to weight stigma by failing to assess “previous attempts at changing a diet.” In contrast, another PCP felt spending even a brief amount of time was beneficial:

*“…even spending 2 or 3 minutes just talking about it with patients, I also think has value. Because I think sometimes just bringing it to the forefront of a patient’s thought processes can also have benefits.”*

Some described nutrition discussions as a “luxury,” at times because patients had more resources. Others, whose practice was described as outside the traditional fee-for-service model, commented on having the good fortune of more time for nutrition. Due to time limitations, PCPs often described time-efficient approaches. This may look like targeting “low-hanging fruit” dietary behaviors they feel are easier changes to make or trying to “hit the highlights.” (Fig 3)

#### Reasons nutrition is discussed.

PCPs mentioned various reasons nutrition is discussed, varying by patient population. For adults, weight- and chronic disease-related conversations were common, either during annual physicals or problem visits, whereas in pediatrics the focus was more on prevention through well child visits along with healthy weight promotion, addressing parental questions/concerns, and picky eating counseling.

PCPs described patients commonly initiating dietary discussions, such as presenting with a nutrition-relevant chief complaint or patients seeking their advice about nutrition topics including what to eat, fad diets, supplements, or nutrition optimization for fitness. Patient-driven weight loss discussions were common, including asking about glucagon-like peptide-1 (GLP-1) therapy. In pediatrics, parents often ask questions about feeding or express concern about body size.

#### Approach to nutrition during patient encounters.

PCPs address nutrition in a variety of ways (Fig 3). Several describe their approach as variable depending on the patient, especially varying by developmental stages in pediatrics, while others describe a method or philosophy employed. How PCPs approach dietary assessment illustrates the variability and inconsistency. PCPs described several methods for assessment that were mostly informal questions, with a questionnaire being less commonly mentioned. A theme in PCPs’ descriptions of addressing nutrition was feeling their approach is fairly general or lacking in detail. Some begin with a general approach and may get into more specifics depending on the patient. The non-specific nature of their approach to nutrition was a common frustration as many feel they are not giving actionable guidance patients can easily implement.

Of PCPs who discussed dietary intervention in relation to pharmaceutical management, some PCPs described prioritizing medication first, especially given time limitations and competing priorities.

*“I’ll spend the bulk of my time explaining…why I care about this illness…talking through what medication options there are…and then why someone’s not taking it…playing around with their insurance to try to get them an affordable medication. And then I feel like I end up trying to squeeze the diet and exercise in there very last…”*

Occasionally PCPs described offering a lifestyle change trial or using nutrition in conjunction with medication. Some commented on medication as protecting patients from disease consequences but lifestyle change as a way for patients to have agency over managing their condition.

*“I try to make this point to a lot of my patients with diabetes that…medicine does not cure diabetes. We’re really just managing secondary risks…At the end of the day, there’s no amount of medicine that I could give that outpaces poor lifestyle decisions, namely with diet. I really try to just set that up…putting it more into their ownership to hopefully recognize that they are in control of this journey, rather than it being on what I am prescribing them…”*

Multiple PCPs described approaches being informed by concern for judgment or stigma about dietary behaviors or weight. Approaches to weight counseling in general were variable (Fig 3). Discussions about weight counseling reflected a tension between an emphasis on weight to address associated health risks and a concern about an overemphasis on weight harming patients. This tension is illustrated by contrasting reflections by two pediatricians. One grappled with concern about detrimental effects of obesity when parents request weight not be discussed:

*“That’s one of the most challenging things I face with this because I feel like, as their doctor, if they weigh 240 pounds when they are 12 years old, there is nothing more important to their health…That is the thing that is going to kill them, to shorten their life, to make their life more miserable right now…if the fact that the parent is saying that, they clearly are already struggling with some body image issues…that’s something that I really struggle with.”*

Another pediatrician struggled with causing harm when discussing weight:

*“I am…aware of all the stigma, shame, and harm around [obesity] and the ways in which…people can have very unhelpful and harmful and shaming conversations, and so I just I try to be very, very, very thoughtful about how I frame all of these discussions…And that’s probably the most challenging thing is the low confidence that I’m going to be able to help instead of harm…”*

#### PCP challenges and frustrations addressing nutrition.

PCPs described several pain points throughout the patient visit and across multiple levels that impact their ability to address nutrition with patients (Fig 3). Themes included time limitations/competing priorities and patient barriers to implementing recommendations. Beyond the clinician and patient levels, PCPs reflected on system-level challenges.

*“…cultivating that interest…in limited time slots and a lot of other things to address is just difficult and it feels like you’re pushing a rock up a hill when the rest of the world has cheap, cheap, low-quality food readily advertised at you 24 hours a day…”*

While participants described frustrations with their training, for some this was not the primary challenge as some PCPs described knowledge as only part of the problem. For example, this PCP describes the difficulties patients face changing dietary habits:

*“I don’t know that…the times where we fail is necessarily because I lack in my knowledge…it’s just the challenges that come with habit…It’s very challenging for patients to change…People are overworked and stressed and it’s just tough sometimes to take the time to…do the things that are needed to do when it comes to nutrition.”*

Due to system-level barriers, some expressed more training in nutrition may not make a difference. As one PCP described, “it feels like too big of a hill to climb.”

#### PCP experience with nutrition support and services.

PCPs reflected on their experience with nutrition support and services, including resources, referrals, and ancillary services to support patients with dietary change. In general, extra support is welcomed but inconsistent.

Whether, how, and what resources PCPs are using were variable with many challenges (Fig 3). Commentaries about resources reflected a gravitation toward ones that are more readily accessible, time-efficient, and easy to conceptualize. Regularity in using resources was variable, with some mentioning resources used often, at times for a specific condition. Preferences varied for using resources in clinic to support counseling versus sending the patient with a reinforcing handout. Examples of resources included visual aids (e.g., healthy plate), handouts on specific diets, and parental guidance resources. Uncommonly, PCPs mentioned resources embedded in the electronic health record system or programs connecting patients with healthy food. Many PCPs described not typically using resources or having limited to no access to them. PCPs expressed multiple frustrations with resources, including dissatisfaction with what they currently use and how much effort is required to find credible resources.

RDs were the predominant referral/ancillary service mentioned. PCPs often praised RDs as a helpful resource, especially for their expertise and time. In general, ease of RD utilization was variable with those with access to RDs in their clinic or health system typically describing their experience collaborating with RDs as easier or more positive (Fig 3). Several PCPs described challenges limiting their ability to rely on RDs for nutrition support, an experience one PCP described as “it ends up falling on me.” Access to RDs was a predominant barrier.

*“…if I place the referral…I’m worried that patients are not actually gonna get the care or support because we don’t have that [nutrition] team as much anymore. I realized that I might be one of the few people that has an opportunity to help support patients with that.”*

Another challenge PCPs commonly expressed was patient receptiveness to or not following through with referrals for a variety of reasons. This PCP describes the value of RD support considering time limitations and competing priorities but the reality that patients may only be willing or able to have a brief discussion with their PCP:

*“In a visit when you’re trying to do medication management, make insulin changes, it’s really hard to have a detailed conversation with somebody about their diet. And that’s where having a separate dietitian appointment is really powerful for folks, but sometimes…they’ll only be comfortable with, or they only have time for the brief intervention you can do right in the room.”*

### PCP perceptions about nutrition in primary care

To capture PCPs perceptions about nutrition in primary care, the following results report on PCP perceptions about the value of nutrition in primary care, satisfaction with and efficacy of their current approach, and easy or exciting aspects of addressing nutrition. The section concludes with perceptions about their role in addressing nutrition as a primary care physician.

#### Value of nutrition.

PCPs often expressed valuing nutrition, calling it “pretty powerful” or an “essential aspect of my patients’ lives.” Many described it as having an important role in primary care, especially for prevention or management of disease or reducing or eliminating medications. While PCPs expressed nutrition deserves more attention in practice and training, in reality it is a lower priority or challenging to address.

#### PCP satisfaction and self-perceived efficacy with their current approach.

When asked how well their current approach is going for them, several expressed it was not going well or they do not feel effective, multiple felt their approach was going “okay” or described a mixed experience, and a limited number described their current approach as going well. Many described efficacy as time- or patient-dependent, but for one PCP, success was measured by follow-through with their guiding framework:

*“I’ve worked out a method where it doesn’t matter to me what the patient brings because I’m not measuring my success by whether they make the change that I’m recommending…I’m just measuring my success about whether I follow through with my principles of things like motivational interviewing…”*

Multiple PCPs described seeing success in a minority of patients, and this was viewed by some PCPs as exciting and by others as not being very effective. Some expressed feeling resigned that “there’s not really much I can do” or that a brief intervention could be more harmful than helpful. This PCP expressed that despite the multiple challenges, at least they are doing something:

*“Unfortunately, around here, the cheapest food is the lowest quality food and sometimes you feel powerless to address that in a clinical setting…it can be really frustrating…So focusing on little things that we can do in the clinic that can actually maybe get patients to change the way they’re doing things is…where we come in and can maybe in some cases actually move the needle just a little bit.”*

#### PCP “delights” in addressing nutrition.

When asked what they find easy or exciting about addressing nutrition, PCPs expressed enjoyment in shared decision-making and relating to patients through conversations about food. PCPs also appreciate conveying the power of nutrition to give patients agency over their health.

*“…something that resonates with me…is…we have control over some of these medical conditions without necessarily using medicine. And so I think patients feel empowered once they realize, hey, just by changing what I eat or changing my approach to certain things, I could potentially reverse this condition or significantly impact this condition, or even come off some medications….We don’t always get a chance to ‘de-prescribe’ medicines.”*

Some PCPs described enjoyment in sharing in patient excitement, success, or “Aha moments,” when patients make the connection that dietary change can help them feel better and improve their health.

#### PCP role in addressing nutrition.

**Fig 4:**
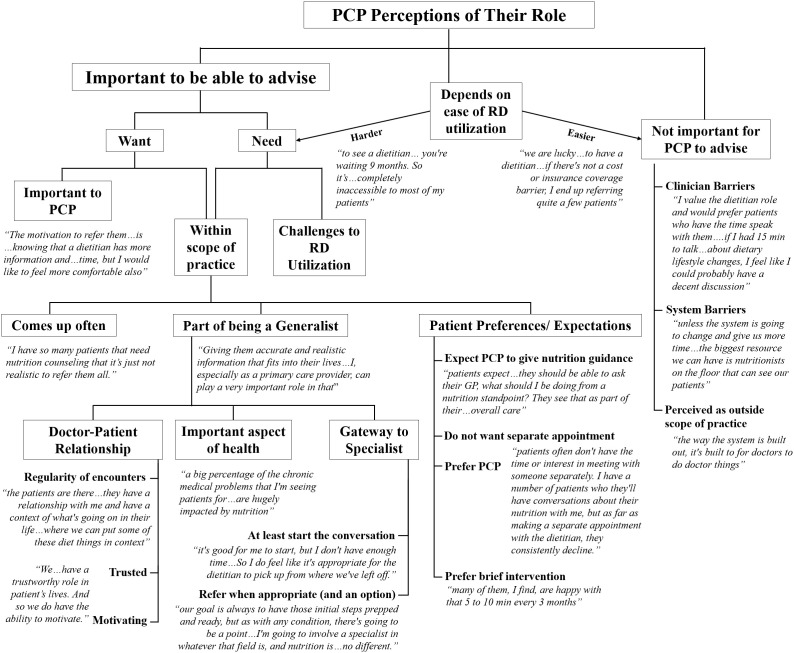
PCP Participant perceptions about their role in addressing nutrition. This schematic outlines the relative importance expressed by PCP participants regarding their ability to comfortably advise their patients about nutrition and a variety of factors influencing their perceptions. The predominant sentiment was that it is important to them to be able to advise in nutrition, reflected by the larger font size. Less commonly, PCPs described the importance as dependent on how easy their experience is with RD utilization or that it is not important for them to advise. A large portion of factors influencing PCP perception place nutrition advising within the scope of their practice. Representative quotes for many of the influencing factors are provided. Abbreviations: PCP (primary care physician), RD (registered dietitian).

PCP perceptions of their role in addressing nutrition varied, and PCPs described multiple factors influencing their role. Fig 4 schematically delineates the variable PCP perceptions of their role, perceived relative importance of PCPs advising about nutrition, and identified factors influencing PCP perceptions of their role. When asked how important it is to them to be able to comfortably advise their patients about nutrition versus referring to a specialist, PCPs overwhelmingly expressed that it is important to them to have the ability to advise patients in nutrition, with rare exception. Reasons for the importance of PCPs advising varied, described as a want, a need, or both. For some, the importance depended on the ease of RD utilization. The following characterizations of the PCP role in addressing nutrition provide examples of the variability of PCP perceptions of their role and illustrate many of the influencing factors outlined in Fig 4.

I *“Fits very well into what being a primary care doctor means to me”*

Some PCPs, regardless of RD utilization challenges, described the need or desire to advise patients in nutrition, often perceiving this as part of their job. Reasons described by PCPs that characterized dietary counseling as within the scope of their practice included patient preferences or expectations, how frequently nutrition comes up, and perceiving nutrition as aligning with their role as a generalist.

*“That’s like our bread and butter is thinking through how we can help keep patients healthy and just develop healthy lifestyles. So I think it’s super important…”*

PCPs also described RDs as the specialist they as generalists could refer to, especially to give patients more time and detailed guidance. The concept of PCPs at least starting the conversation about nutrition, whether or not RD support was an option, emerged as a theme.

II *“They may only have me”*

RD utilization challenges were a common reason PCPs felt it is important for them to comfortably advise their patients about nutrition.

*“…a lot of times when we put the referral in, it either never goes through, as in the patient never schedules the appointment, or they go once and they don’t go back because it was a cost-limiting factor. And so I think it’s really important from a primary care perspective that I have some tools and education to be able to provide that…I think the barriers to having that done consistently are high enough that being able to do it in primary care makes a lot more sense.”*

III *“Not enough time just to talk about medical things”*

Some PCPs described dietary counseling as not a good use of their time due to health system barriers.

*“I think in my ideal way that our medical system would work and the format that I would like to practice in, it would be really great if I had greater confidence and ability there. I think the reality of our system as everyone’s getting more specialized, and also, frankly, patients tend to get more sick, I don’t see getting to be able to do that where it makes sense with the time I have available with my patients.”*

Uncommonly, PCPs characterized nutrition as outside the scope of their practice, especially given how the health system is structured with PCPs facing competing priorities and time constraints.

IV *“It’s both referring and talking about it myself”*

Several PCPs described their role in nutrition as a balance of advising and referring, with PCPs and RDs having distinct and important roles, often with the PCP getting the “ball rolling.” However, despite many PCPs wanting to involve RDs, at times that brief intervention starting the conversation is the only option for patients due to RD utilization barriers. Adding to the complexity, some PCPs expressed concern about patients receiving conflicting recommendations and wanting to be on the same page with RDs.

*“…if the question is how important is it for me to be able to address it period, that’s super important. If it’s I wanna do it, and I don’t want nutritionists involved, I don’t think that’s the case. Again, I would love for that resource to be more utilized and for us to find a way to be on the same page about that. I’m not opposed to working on that hand in hand.”*

PCPs recognize their role is distinct from RDs but also value addressing nutrition themselves.

*“I know that I’m not a dietitian, but I would just like to have [dietary counseling] be a consistent part of my visits with patients, too.”*

### Step two: Defining the problem

**Fig 5:**
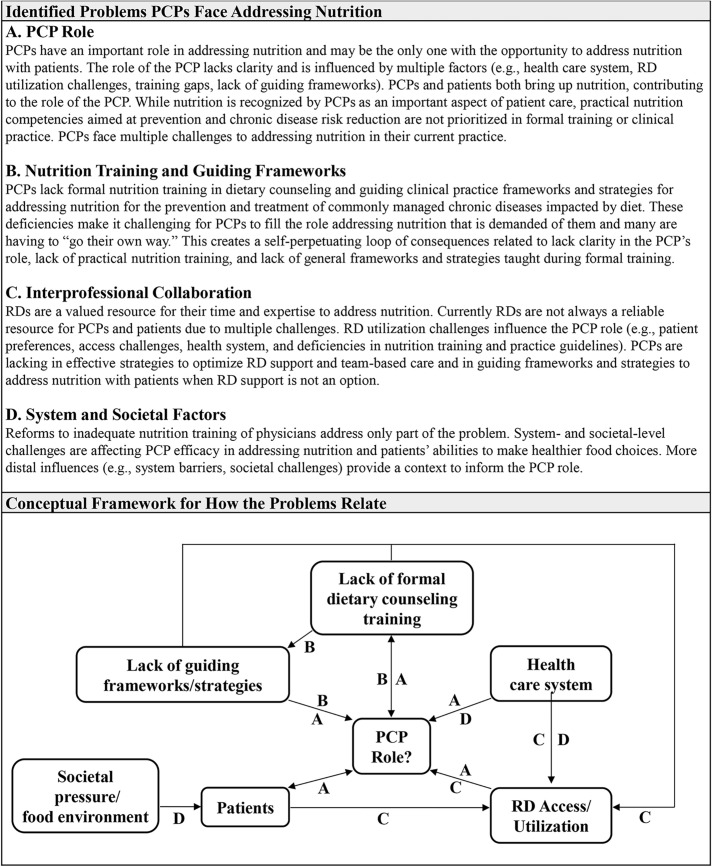
Identified problems and conceptual framework. Fig 5 lists the identified problems faced by PCP participants in addressing nutrition based on analysis of interview findings and schematically shows their relationship. Abbreviations: PCP (primary care physician), RD (registered dietitian).

In continuing the design thinking process (Fig 1), after empathizing with PCPs, four overlapping problems were identified. The interconnected problems are listed and schematically represented in Fig 5.

## Discussion

Results from this qualitative study add to the literature a comprehensive understanding of PCPs’ experiences addressing nutrition that spans training, clinical practice, and interprofessional collaboration. The breadth of the research question and the depth of the interviews provide a holistic view of the primary challenges PCPs experience and how these problems relate. In line with the design thinking framework of understanding the problem before ideating solutions, end-user needs based on the corresponding problems in Fig 5 are stated and discussed.

### PCP needs to improve their experience addressing nutrition

#### A. PCP role: PCPs need clearly defined expectations for their role as a generalist in addressing nutrition that prioritizes the importance of nutrition in their patients’ health and matches the reality of clinical practice demands.

Despite how often PCPs are addressing nutrition, whether patient- or physician-initiated, the role and responsibility of PCPs in addressing nutrition lacks clarity, as Fig 4 demonstrates. Findings from this study support nutrition as within the scope of primary care practice for physicians to address in routine patient care, and dietary guidance as a means to prevent or treat disease already aligns with the definition of medical practice set by the Federation of State Medical Boards [43]. PCPs value nutrition, and patients expect their physician to advise them in nutrition. While they value the expertise of RDs, when available, PCPs also recognize they may be the only ones to counsel patients about diet given RD utilization challenges, a sentiment previously shared by medical trainees [35]. RD access limitations have been reported by other PCPs [44]. While PCPs expressed that nutrition is an important aspect of primary care and that they can motivate patients to make dietary changes, they repeatedly conveyed the low priority of nutrition in training and clinical practice, characterized in both domains as getting pushed to the end, glossed over, or lacking in specifics to give practical guidance to patients. The lack of prioritizing nutrition, especially in clinical curricula, may influence trainee attitudes about their role in dietary counseling by signaling to trainees that nutrition is outside the scope of their practice [45]. In systems-based medical curricula [46], integrating nutrition throughout presents challenges to prioritizing nutrition as nutrition influences multiple organ system, such as cardiovascular [47] and endocrine [48], and subsequently nutrition may be “sprinkled” throughout, as one PCP described. How PCPs are accessing nutrition resources for use in practice reflects a similar pattern of resources not concentrated in any one easy-to-find place.

Another reason nutrition may be less prioritized is that PCPs have clear roles in diagnosis and treatment, reflected by PCPs prioritizing “medical things” or “doctor things.” PCPs have responsibilities that other team members cannot take on, and it makes sense to delegate nutrition care, deserving of more time and attention that RDs are equipped to provide, to the nutrition expert. However, current challenges with RD utilization make this only an option for some patients, and, with the frequency that nutrition comes up, PCPs need to be equipped with frameworks and strategies to discuss diet with patients that they can implement regularly. Additionally, while the RD is the nutrition specialist, dietetics is a profession and training path outside of the traditional medical specialization trajectory of medical school, residency, and fellowship that is familiar to physicians and that promotes learning from and regular interaction with the specialist. The lack of overlap in training paths could contribute to silos between dietetics and medicine, which may hinder collaboration and contribute to a *discontinuity* of care with nutrition, as PCPs expressed concern about patients receiving conflicting advice.

#### B. Nutrition training and guiding frameworks: PCPs need formal training in guiding frameworks and strategies to approach the prevention and management of common diet-related chronic diseases that aligns with a clearly defined and realistic role in addressing nutrition with patients.

Findings align with the longstanding recognition that physicians are inadequately trained in nutrition [9,10,45,49], despite patient expectations [50]. Recent cross-sectional studies document no evidence of improved nutrition knowledge among PCPs more proximal to training compared to more distal [51] and no association between how often physicians give nutrition advice and how long they have been in practice [52], suggesting a continued need to consider and address how physicians are trained in nutrition. Results from this study add to the picture a deeper understanding of the consequences of deficiencies in formal nutrition training in medicine, namely that physicians are resorting to learning nutrition knowledge they need on their own in informal, ad hoc ways. PCPs are already busy, adding to the challenge of learning and keeping up to date with nutrition. Deficiencies in nutrition training and subsequent self-directed learning also contribute to variations and inconsistencies in recommendations and approaches, as PCPs are not being trained in guiding frameworks and strategies for dietary counseling, particularly in dietary counseling for risk reduction related to chronic diseases they are commonly managing. For example, an area demonstrating a need for formal training in a guiding evidence-based and health-promoting framework is weight counseling, given the tension expressed by PCPs trying to promote health and minimize harm, as weight stigma in health care and how physicians are trained in weight management are known problems [53–55].

Aligning with the variability of approaches reported by PCPs in this study, medical trainees have expressed frustration with lacking a guiding framework for dietary counseling [35]. Fig 5 depicts a self-perpetuating loop between the lack of formal training, lack of guiding frameworks/strategies, and lack of clarity in the PCP’s role in addressing nutrition. These deficiencies are especially notable for commonly managed chronic diseases. Clearly defining the role of the PCP can help break this cycle and inform guiding dietary counseling frameworks and strategies for the generalist and subsequently help distill realistic and priority nutrition competencies for physicians to be taught in a more systematized way. Moreover, establishing guiding frameworks for dietary counseling for health promotion and chronic disease risk reduction for the generalist in primary care can serve as a benchmark for PCPs to measure success, judging themselves based on whether they follow through with the guiding principles expected of them, which in turn can improve PCP confidence and satisfaction addressing nutrition. Given the predominant pain point of patient motivation, assessing patient readiness to change and motivational interviewing (MI) are important skills physicians are introduced to in medical training and ones that can be harnessed for dietary counseling training as it helps physicians recognize readiness to change, align expectations accordingly, and work more effectively in tandem with RDs. MI can also reduce shame and stigma in addressing dietary behaviors [55]. While RDs are highly skilled in MI [56], it is often the PCP who sees patients in the early stages of readiness to change, and MI may even be useful for motivating patients to visit with RDs for more in-depth support.

#### C. Interprofessional collaboration: PCPs need improved strategies to more effectively collaborate with dietitians to support patients with nutrition care, clarified guidance for referring patients to the nutrition specialist, and practical frameworks and strategies for addressing nutrition as the generalist, especially when RD support is not an option.

Despite the interconnectedness between nutrition and health, results suggest dietetics and medicine are relatively siloed professions, beginning in medical training as previously discussed. In a recent survey of family physicians, 64% of participants reported not having an on-site RD [44], making it more challenging for PCPs and RDs to benefit from face-to-face interaction and regular communication that support a collaborative working relationship [57]. Disconnect is also reflected in our findings regarding PCP experiences with RD collaboration, such as PCPs lacking awareness of what RDs are recommending and expressing concerns about being “on the same page.” PCPs have previously reported uncertainty surrounding RD support and how to locate RD services as a barrier [44], while another study documented an appreciation for consistency and “having a shared language” in interprofessional team care [58]. Interprofessional education (IPE) and team-based care are emphasized in accreditation standards during medical training [33,59]; however, PCPs infrequently mentioned RD involvement in their formal training. In clinical practice, a clear understanding of roles is vital to fostering collaboration in interprofessional team-based care [57]. Clarity in expectations of physicians and subsequently in the dynamics of the physician-dietitian team approach can inform how RDs teach medical trainees through IPE and could improve RD utilization by supporting physicians with guidance for getting the “ball rolling,” making referrals, and more effectively endorsing RDs.

Given that results from this study support dietary counseling as within the scope of primary care practice, conceptualizing nutrition care as a spectrum of PCPs at least initiating dietary discussion and focusing on high-yield recommendations then referring to the specialist when appropriate may add clarity to the PCP’s role and improve RD utilization. In a recent survey, health care providers, the majority physicians, reported advising about nutrition more than referring to RDs for wellness promotion and chronic disease conditions, supporting the concept of PCPs as the generalist in the spectrum of nutrition care [52]. RDs have the time and expertise while PCPs have the longitudinal and trusted relationship with more opportunities to engage with patients on a regular basis. A shift from non-overlapping roles to a spectrum of team-based nutrition care would be beneficial as flexibility is needed based on what patients are willing and able to do, whether it’s a brief intervention with their PCP during a routine visit or RDs available in clinic for co-visits at no extra cost to the patient. Formal training of physicians in guiding frameworks and strategies for dietary counseling could support not only PCPs in those initial discussions and in brief dietary counseling but also patients in receiving continuity of nutrition care when seeing the specialist. Given that RD support is not always an option, general frameworks for addressing nutrition in primary care would better equip PCPs to support their patients in having greater agency of their health through diet.

Simultaneously, reforms to RD access are needed as PCPs repeatedly expressed frustration with the cost and insurance barriers to accessing RDs, a top barrier reported by PCPs previously [44], while those with RDs in their clinic described easier collaboration. RDs are increasingly more accessible for telehealth visits [60], which may add helpful flexibility for patients. Patient preferences for brief dietary discussions and not wanting to have a separate appointment are potential factors to consider in exploring and designing strategies to better utilize the valuable support RDs provide, such as through the MD-RD co-visit model that one participant described. Innovative models for dietetic support may help improve RD utilization, such as shared medical appointments with RDs [61], culinary medicine integration in RD support [56,62]. or RDs engaging with patients shopping for groceries [63].

#### D. System and societal factors: PCPs need frameworks, strategies, and improved resources for addressing nutrition within the context of system and societal barriers and for these barriers to be thoughtfully addressed through policy, systems, and environmental change strategies.

Multiple calls to action to address inadequacies in nutrition training in medicine address a critical and urgent need [9–14]; however, the results from this study highlight the need to simultaneously address system- and societal-level challenges. While PCPs continue to report that they need more formal training in nutrition, particularly in practical dietary counseling competencies, nutrition knowledge was not always the top concern compared to system- and societal-level barriers, an experience shared by other PCPs [20]. Limited time was a running theme as PCPs shared their experiences trying to fit in nutrition, in line with prior sentiments shared by PCPs over the past few decades [15,17,20,21,23], and this was related back to the health system and patient panel expectations. Of note, participants practicing outside the traditional fee-for-service model relished the extra time and flexibility to make nutrition more of a focus. The relationship between primary care delivery through alternative primary care models, such as value-based care and direct primary care, on quality of nutrition care is deserving of further exploration. Many RD utilization challenges related to health system barriers, such as insurance coverage constraints and the cost to patients as well as funding barriers to support RDs on staff, suggesting a need for health services, health payer, and policy reforms. Another predominant challenge was the impact of the food environment on patients’ abilities to make dietary changes, including the cost of food and an overabundance of inexpensive, ultra-processed foods readily accessible to patients that at times made PCPs feel powerless to effect change. Simultaneously, few PCPs reported offering resources connecting patients with food, such as Food is Medicine interventions, perhaps reflecting underutilization of a resource addressing many barriers patients face trying to follow their physician’s recommendations [64].

### Strengths and limitations

The qualitative design, broad research question, and human-centered design approach allowed for an in-depth and comprehensive exploration of PCPs’ experiences addressing nutrition with patients. As such, the results contribute a deeper understanding of a complex problem and how many of the challenges relate, rather than exploring more focused aspects of PCP experiences individually. The research question and study sample for this study were intentionally broad in looking at the holistic experience of PCPs that allowed for understanding of PCP experiences within individual patient encounters and longitudinally across patient visits. The breadth of the study also captured PCP experiences across the patient life course by including pediatricians, internists, and full spectrum PCPs as overlapping experiences and needs exist. Findings also extend beyond the patient visit by capturing PCP experiences with resources and referrals.

While originally intending to employ purposive sampling to increase the variety of PCP perspectives, not enough PCPs completed the scheduling process for this to be needed. We interviewed all PCPs who completed the scheduling process as data saturation was not reached, likely due to the broad inclusion of PCPs based on patient population and clinical practice settings and the duration of interviews. Those with greater interest in nutrition or greater frustrations with dietary counseling may have been more likely to participate; however, this may provide richer data about approaches and challenges to addressing nutrition in primary care. Results are limited in generalizability, particularly related to nutrition training experiences, given the participants were all UNC SOM graduates, and this may influence participant attitudes about nutrition as attitudes are a component of competencies in medical education. However, the general sentiment that formal training in nutrition is inadequate is widely acknowledged in the literature, and medical trainee frustration with lacking training in counseling strategies has been reported elsewhere [35]. The racial and ethnic representation of participants was fairly homogenous, also limiting generalizability and breadth of participant experiences included in this study. Despite recruitment limitations, participants reflected a variety of fields, practice types, locations, and patient populations. Family physicians were the predominant field participating; however, as physicians trained in full-spectrum primary care, they provide a rich perspective on nutrition bridging pediatric and adult primary care. Seeking participant feedback on the conceptualization of the problems identified would have strengthened this study. Due to limitations in generalizability, a future direction of this study is to corroborate findings from the qualitative data on a larger scale through quantitative methods. Another future direction is to continue the design thinking process (Fig 1) by exploring potential solutions, including analyzing data relevant to ideal situations and facilitators of addressing nutrition described by PCPs in the interviews and prototyping and iteratively testing solutions with end users.

## Conclusion

While findings from this study support dietary counseling as within the scope of the PCP’s practice, the responsibilities that realistically can be expected of generalists in routine primary care visits need clarity, especially given the number of challenges and frustrations expressed by PCPs across multiple levels. PCPs recognize they need to be able to address nutrition, but gaps in nutrition training lead them to learn many practical nutrition competencies on their own, figure out their own approach, and try to find helpful resources. PCPs, who rely on medical school and residency to train them in the competencies they need for clinical practice, lack a clearly defined role in addressing nutrition. This role needs to be clarified and not only taught in a formal, systematized manner but also supported with guiding frameworks and strategies and easier access to evidence-based resources. PCPs also need to be supported in their role in nutrition through addressing health system barriers, especially time constraints and RD access challenges. The defined problems and needs identified provide foundational knowledge to build on in exploring potential solutions, subsequent steps in the design thinking process. Finally, while nutrition training reforms advance, researchers, health payers, and policymakers need to simultaneously consider and address why nutrition is losing out to competing priorities and getting pushed to the end of patient visits despite the power of nutrition to promote wellness and manage many of the most common chronic diseases.
